# Design, construction, and deployment of a multi-locus transcranial magnetic stimulation system for clinical use

**DOI:** 10.1186/s12938-025-01393-6

**Published:** 2025-05-18

**Authors:** Heikki Sinisalo, Olli-Pekka Kahilakoski, Victor H. Souza, Jaakko O. Nieminen, Robin Rantala, Timo Tommila, Isabel Usuga, Mikael Laine, Oskari Ahola, Eva Gallegos, Gábor Kozák, David Emanuel Vetter, Ilkka Rissanen, Andreas Jooß, Renan Matsuda, Ana M. Soto, Dezhou Li, Dania Humaidan, Matti Stenroos, Timo Roine, Dubravko Kičić, Ulf Ziemann, Risto J. Ilmoniemi

**Affiliations:** 1 Department of Neuroscience and Biomedical Engineering, Aalto University School of Science, Espoo, Finland; 2 Department of Neurology & Stroke, University of Tübingen, Tübingen, Germany; 3 Hertie Institute for Clinical Brain Research, University of Tübingen, Tübingen, Germany

**Keywords:** Transcranial magnetic stimulation, mTMS, Functional imaging, Motor mapping, Patient safety

## Abstract

**Background:**

Transcranial magnetic stimulation (TMS) is an established method for noninvasive brain stimulation, used for investigating and treating brain disorders. Recently, multi-locus TMS (mTMS) has expanded the capabilities of TMS by employing an array of overlapping stimulation coils, enabling delivery of stimulation pulses at different cortical locations without physical coil movement. We aimed to design, construct, and deploy an mTMS device and a five-coil array for clinical environment, emphasizing safety of the system.

**Methods:**

Our mTMS device is controlled by a field-programmable gate array (FPGA). The power electronics comprises five stimulation channels, each consisting of a high-voltage capacitor connected to a pulse circuit, controlling a single coil in the array. The device contains custom-designed circuit boards, with functions such as monitoring the system state, reporting errors, and delivering pulses. Our design utilizes redundancy in both hardware and firmware to ensure robust operation and safety. We performed an automated motor mapping test to verify the electronic targeting capabilities of the device.

**Results:**

We constructed the mTMS device and deployed it to the Hertie Institute for Clinical Brain Research (Tübingen, Germany). Compared to our earlier prototype, the new design improves patient and operator safety. The motor mapping test confirmed that our device can accurately target stimulation pulses in the cortex.

**Significance:**

mTMS or other similar technologies are currently not available for hospital use. The present device and its installation are major steps toward establishing multicoil TMS as an accessible clinical tool for investigation and treatment of the brain.

**Supplementary Information:**

The online version contains supplementary material available at 10.1186/s12938-025-01393-6.

## Introduction

Transcranial magnetic stimulation (TMS) is a widely used noninvasive brain stimulation method [1] for research [2], diagnostics [3], functional imaging and mapping in neurosurgery [4–6], and treatment of various diseases, including depression and chronic pain [7, 8]. Recently, there has been a growing interest in stimulating multiple cortical sites with interstimulus intervals in the range of milliseconds. This would allow rapid imaging protocols, leading to higher clinical throughput and cost-effectiveness. In addition, such capability enables investigating functional networks in the brain [9–11], potentially leading to novel imaging methods and improved treatment outcomes [12, 13].

With conventional TMS, it is difficult to target nearby cortical areas with subsequent pulses in the millisecond scale, as physically moving the stimulation coil is slow, even with the assistance of robotic platforms [14–16]. We have recently introduced multi-locus TMS (mTMS) technology, allowing electronic targeting of cortical loci without physically moving the coil [17]. In mTMS, multiple TMS coils with distinct winding patterns are stacked on top of each other, partially or completely overlapping. Each coil induces its own electric field profile on the cortical surface. These profiles can be combined to form a range of field patterns on the cortex by driving multiple coils simultaneously. By adjusting the current in each coil, the resulting focal region of the induced electric field pattern can be electronically shifted or otherwise manipulated [18]. This way, the stimulus location and orientation can be adjusted with millimeter resolution. With mTMS, the interstimulus interval can be as short as a millisecond or less, even when targeting separate locations with different intensities [19–22]. The capabilities of mTMS have been demonstrated on healthy human participants [17–19, 23–27] and in a preclinical setting with simultaneous magnetic resonance imaging [28]. Furthermore, Tervo et al. [29, 30] designed an algorithm to automatically find optimal stimulation locations and orientations based on electromyography (EMG) and electroencephalography (EEG) readouts.

At the time of writing, a few alternative approaches to implement multicoil TMS have been proposed [18, 31–33]. However, none have yet matured into fully functional, easy-to-use systems for clinical applications. Translating a prototype of a medical device into clinical use typically involves regulatory compliance, safety and risk management, manufacturing, quality assurance, and extensive documentation. Thus, the clinical utility and feasibility of targeting and modulating functional networks in the brain, e.g., by using tractographical priors [34, 35], remains to be addressed, as clinical installations have been lacking.

Although our earlier device [18] can be used for such investigations, it requires a high level of expertise due to its prototypical nature. A key safety feature currently missing is a robust state-tracking mechanism. Such a mechanism would keep track of the system’s operational status via distributed, redundant safety checks, with inconsistencies resulting in disarming the device. An mTMS device suitable for hospital use would enable clinical trials and extensive research on new therapeutic and diagnostic protocols, including study of mechanisms related to network brain functions.

The goal of this work was to develop and install an mTMS system compliant with electric and operational safety standards for clinical environment. In the design, we focused on the robustness of the system and took preventative measures to ensure patient and operator safety, similar to the requirements of commercial systems. We consider this a critical step toward establishing and validating electronic targeting of TMS in patients and developing diagnostic or therapeutic paradigms.

## Results

The mTMS system was successfully installed in the Hertie Institute for Clinical Brain Research in Tübingen, Germany.

### Verification of multicoil targeting

**Fig. 1:**
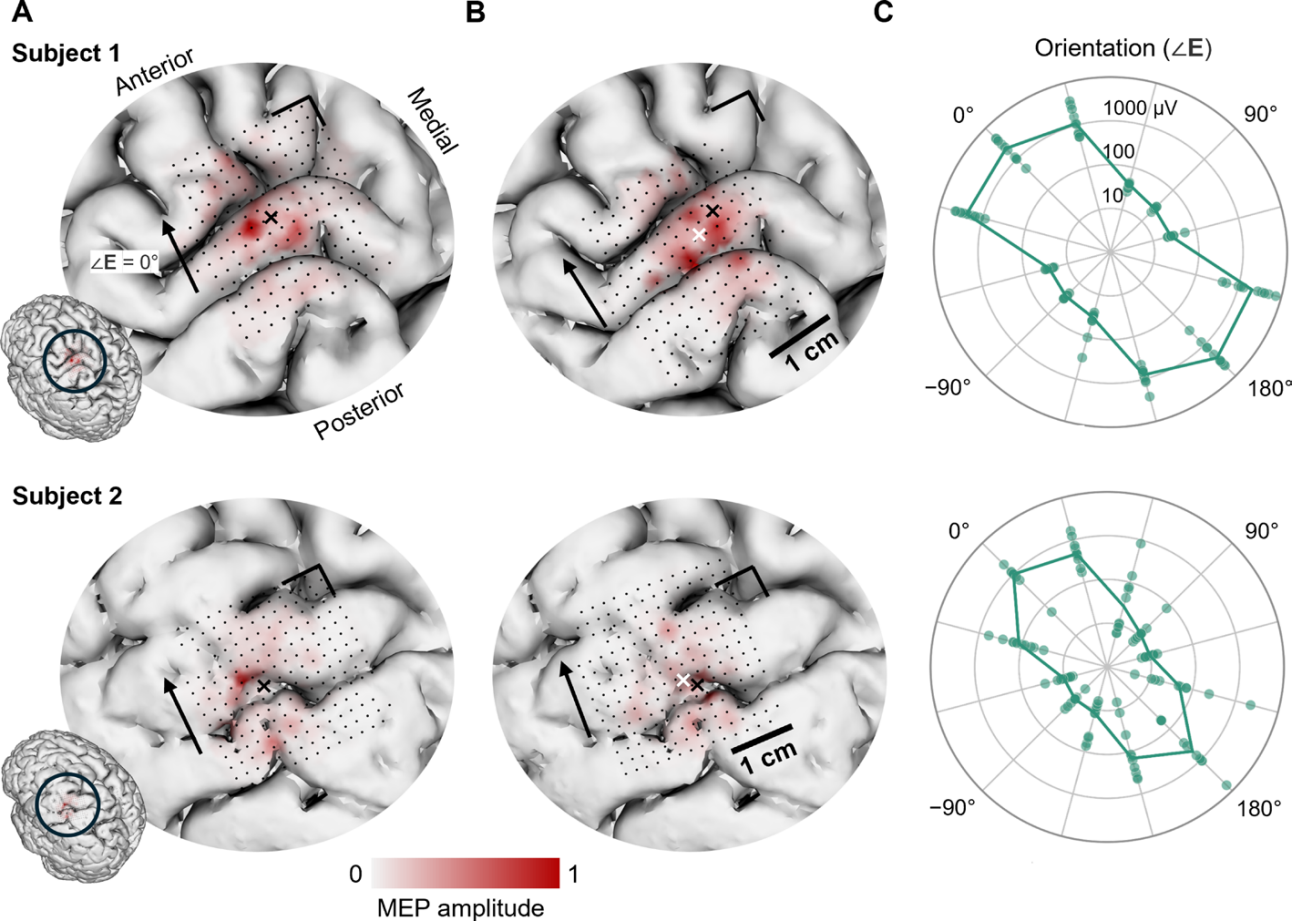
Spatial and orientation response graphs for both subjects. Normalized MEP amplitudes as a function of 2-dimensional electronic shifting of the stimulation target before (**A**) and after (**B**) moving the coil array. The black and white crosses mark the weighted centroid before and after the movement, respectively. The black arrow indicates the zero angle of the peak electric field. The black line forming a right angle serves as an anatomical reference. A Gaussian spatial filter was applied to the data to remove noise and highlight the areas of maximal response. (**C**) The orientation-dependent MEP response at the centroid (white cross) after moving the coil array in 30-degree increments

Physically moving the coil array kept the area of the highest MEP responses relatively intact but also revealed motor responses on parts of the brain surface that were not obtained with the original placement of the coil array (Fig. 1A, B). The centroids of the MEP responses moved by 4 mm and 3 mm in grid coordinates for the two subjects, respectively.

The orientation response mapping indicates that stimulating with zero angle, i.e., roughly perpendicular to the precentral gyrus, maximized the motor response. A second maximum occurred at a 180-degree angle, in line with previous studies [23, 56] (Fig. 1C).

### Temperature estimation

Despite being digital, the temperature sensors integrated into the coil array functioned well in the presence of the strong electromagnetic interference. However, we discovered that the sensors were located too far away from the point of contact to the subject’s head. Consequently, the sensors could not be used to reliably estimate the rapid temperature changes on the center point of the coil array’s surface.

Therefore, during the test, we periodically measured the temperature with a thermal camera (TG165-X; Teledyne FLIR LLC, USA) to ensure that the coil array’s surface in contact with the subject’s scalp did not exceed the safety limit of 41 °C.

## Discussion

We designed, built, and deployed a 5-channel mTMS device and an accompanying 5-coil array for a clinical environment. The device and the coil array comply with the applicable medical device standards to guarantee their safety and reliability. The design improves upon our previous mTMS devices [17, 18, 28]. We implemented a robust state-tracking mechanism, which assesses the operational state of the device based on distributed safety checks and heartbeat messages, quickly disarming the device after a malfunction or other discrepancy. In addition, the coil array was designed to have two means of patient protection to shield the subject against coil breakage. The device was successfully installed at the University of Tübingen, Germany, in October 2022.

We performed extensive tests on the hardware, including confirming that the amplitudes and directions of the electric currents through the coils were as intended. Furthermore, we verified the electronic targeting capability of the system by performing a motor mapping test. The motor map was placed in such a way that it contained the hand knob area and the FDI hotspot.

The motor mapping test verified that our mTMS device can stimulate distinct cortical locations at various orientations using electronic targeting. The peak response areas differed slightly between the response maps obtained before and after moving the coil array (Fig. 1A, B). This could be due to several factors: (1) the spherical head model used for generating the targeting grid does not account for the convoluted morphology of the cortex and cerebrospinal fluid, (2) the coupling of the coils to the brain can differ depending on the coil array’s location and tilt, and this cannot be reliably estimated with the spherical model used, (3) due to the coil array’s different tilt before and after the movement, the stimulation may have affected areas outside the intended target, potentially with a different relative orientation than the one found for the hotspot, and (4) inherent measurement variability and noise. Further studies can elucidate the factors affecting cortical mapping with electronic targeting.

The mapping of the orientation response further supports our hypothesis that the targeting works as intended. The response was strongest with the posterior–anterior rather than anterior–posterior electric field direction on the cortex, gradually diminishing when rotated to align with the central sulcus. This result agrees with previous studies and literature [23, 56].

In the future, we plan to revise the centralized FPGA-based architecture, moving toward a more distributed design. Whereas an FPGA is excellent for small-scale prototyping, its major drawback is the limited availability of resources, especially with larger designs [57]. In addition, FPGA development is challenging due to its low-level nature and concurrent operation [58]. The slow development cycle [59] and inherent non-observability [60] of the FPGA complicate finding and resolving programming errors. In the current design, these shortcomings were partly addressed by distributing the internal control of the device to the other programmable modules.

In our motor mapping test, the suboptimal locations of the temperature sensors in the coil array prevented reliably estimating its temperature. However, it is difficult to place the sensors close to the center of the coil array due to the dense coil windings, warranting further investigation. In multicoil TMS systems, it is also vital to incorporate efficient cooling strategies, which are currently limited in our design. This is particularly important when performing rTMS or multipulse protocols, introducing potential new challenges.

Our mTMS device allows using any combination of up to five coils, provided that their inductances and resistances are compatible with the power electronics of the device. For example, coil arrays can be designed to enable stimulation of larger areas [61] or distinct brain regions. Additionally, nonoverlapping or multiple-axis coils [32] can be used.

We believe that it is important to make multicoil TMS systems available to neuroscientists and clinicians, as rapid electronic targeting of stimulation offers new kinds of opportunities for research, treatment, and diagnostics [11, 62]. In particular, neurosurgical procedures [4–6] could greatly benefit from rapid, operator-independent, and streamlined imaging protocols. In this early stage, we plan to develop the present system further in cooperation with the on-site researchers, while collecting valuable data on its functionality. In addition, we will use the mTMS device to develop stimulation paradigms that were previously unattainable, including simultaneous stimulation of two- or three-node networks and real-time, closed-loop control of stimulation parameters.

## Conclusion

In this paper, we present our mTMS device designed, built, and employed for clinical environment. We anticipate mTMS to increase patient throughput and enable the exploration of more efficient non-invasive neuromodulation treatments.

## Methods

In this section, we describe the requirements for the installation site, the components of the mTMS system, focusing on its safety features, and the software and firmware used in the device. Additionally, we describe how its functionality was verified.

**Fig. 2:**
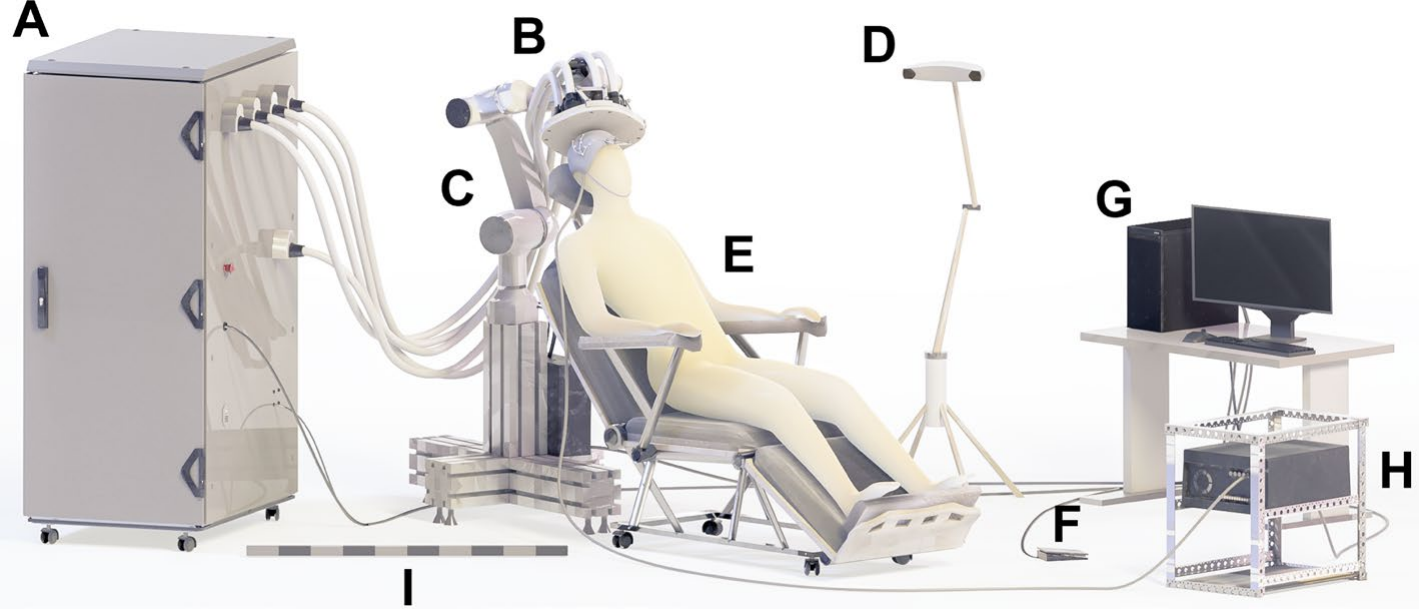
The components of the system: **A** mTMS cabinet, **B** coil array and trackers, **C** robotic arm, **D** stereo camera for navigation, **E** patient chair, **F** configurable foot pedal, **G** PC with control software, **H** data acquisition system, **I** size scale of 1 m

The system includes two major parts: (1) the mTMS device, i.e., the physical cabinet, containing the power electronics and control logic (Fig. 2A), and (2) an array of overlapping coils (Fig. 2B), capable of electronically adjusting the location of stimulation within a cortical area of 30 mm in diameter. The coil array is moved and held in place by a collaborative robot (Elfin 10; Han's Robot Co Ltd, China), guided by an open-source robot control software [25, 26]. An open-source neuronavigation software (InVesalius 3) [36] is used with an infrared camera (Polaris Vicra; Northern Digital Inc, Canada) to provide both visual feedback to the operator and position information for the robot control. A commercial TMS-compatible EEG system (NeurOne; Bittium Corp, Finland) is used to record EEG and EMG data. In addition, the setup includes a PC for preparing and running the experiments. The PC runs custom software to control the mTMS device. Figure 2 illustrates the components of the system installed in a laboratory environment.

The system is intended for exploratory research, including real-time closed-loop stimulation protocols, in which stimulation is algorithmically adjusted based on data from EEG, EMG, or other sources. The pulse waveforms can be adjusted arbitrarily, allowing submillisecond interstimulus intervals when targeting different cortical locations [19–22].

### Site requirements

Deploying an mTMS system imposes several requirements for the installation site. Compared to typical commercial TMS devices, the mTMS cabinet is larger, with dimensions of 0.65 m × 0.8 m × 1.5 m. Additional space is required for the patient chair, the robotic arm, and the control PC, whereas enough space should be reserved for patient preparation. Furthermore, the considerable weight (ca. 200 kg) of the cabinet needs to be accounted for when planning its delivery and assembly. The neuronavigation for the mTMS system can follow a typical clinical setup with a movable camera on a stand, requiring its own space. Optionally, a multicamera setup with fixed cameras on the wall [25, 26, 37] can be used, freeing up floor space. Measurement instruments, such as the EEG device, may benefit from a location away from the walls to reduce potential electromagnetic interference [38–40]. For the mTMS device, standard single-phase power (16 A, 230 V) is sufficient.

The location of the laboratory should be considered carefully. For instance, TMS pulses can affect sensitive instruments or life-supporting devices. In addition, the room should be acoustically isolated from the environment: the stimulation coils produce loud impulse noise [41] and the experiments should not be disturbed by external sounds. Acoustic panels can control echoes and sound reverberation in the room. Removing visual distractions such as windows should be considered. Finally, enough extra space should be reserved for people to operate conveniently.

### Device composition

**Fig. 3:**
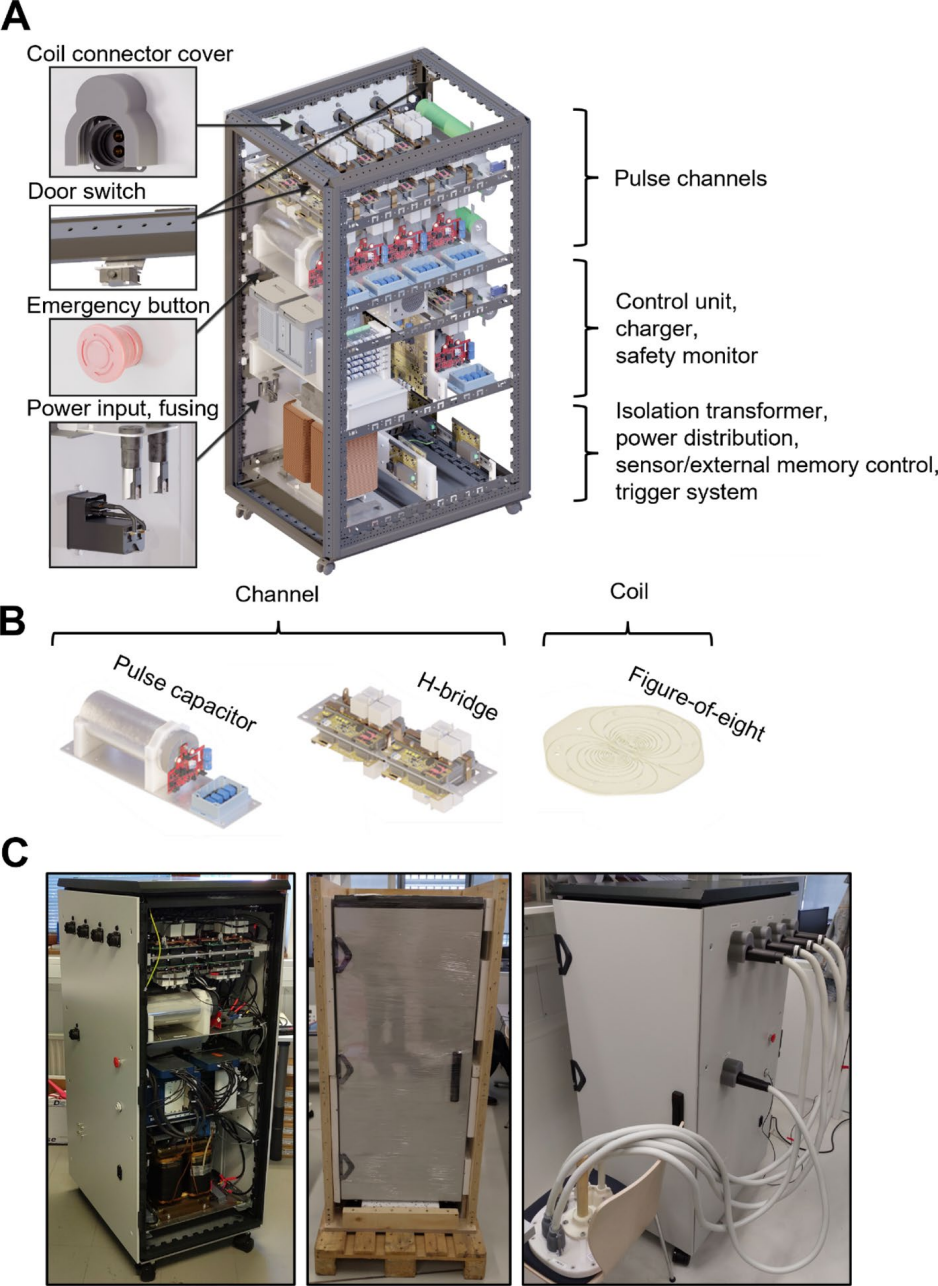
**A** schematic of the mTMS power electronics cabinet. **B** an individual channel consisting of the electronics required to drive the TMS pulse and the stimulation coil. **C** Photographs of the device. Left: with front door removed. Middle: after transportation. Right: final installation with coil array connected

Our mTMS device is divided into five separate, independently operating stimulation units, with each unit controlling one stimulation coil. The units are housed inside a grounded, air-cooled cabinet (Varistar; Schroff GmbH, Germany), shown in Fig. 3A. The coils are encased inside a protective enclosure, and they can be driven concurrently to enable electronic control of the induced electric field pattern in the cortex.

The overall design improves upon our previous work [18]. In particular, the safety features of our prior device were evaluated and revised, leading to multiple changes to the circuit board modules. Furthermore, major revisions were made to the software, including the firmware running on the microcontroller units (MCUs) and the interface to the control PC. The power electronics remained largely the same, except for minor differences in the physical layout.

**Fig. 4:**
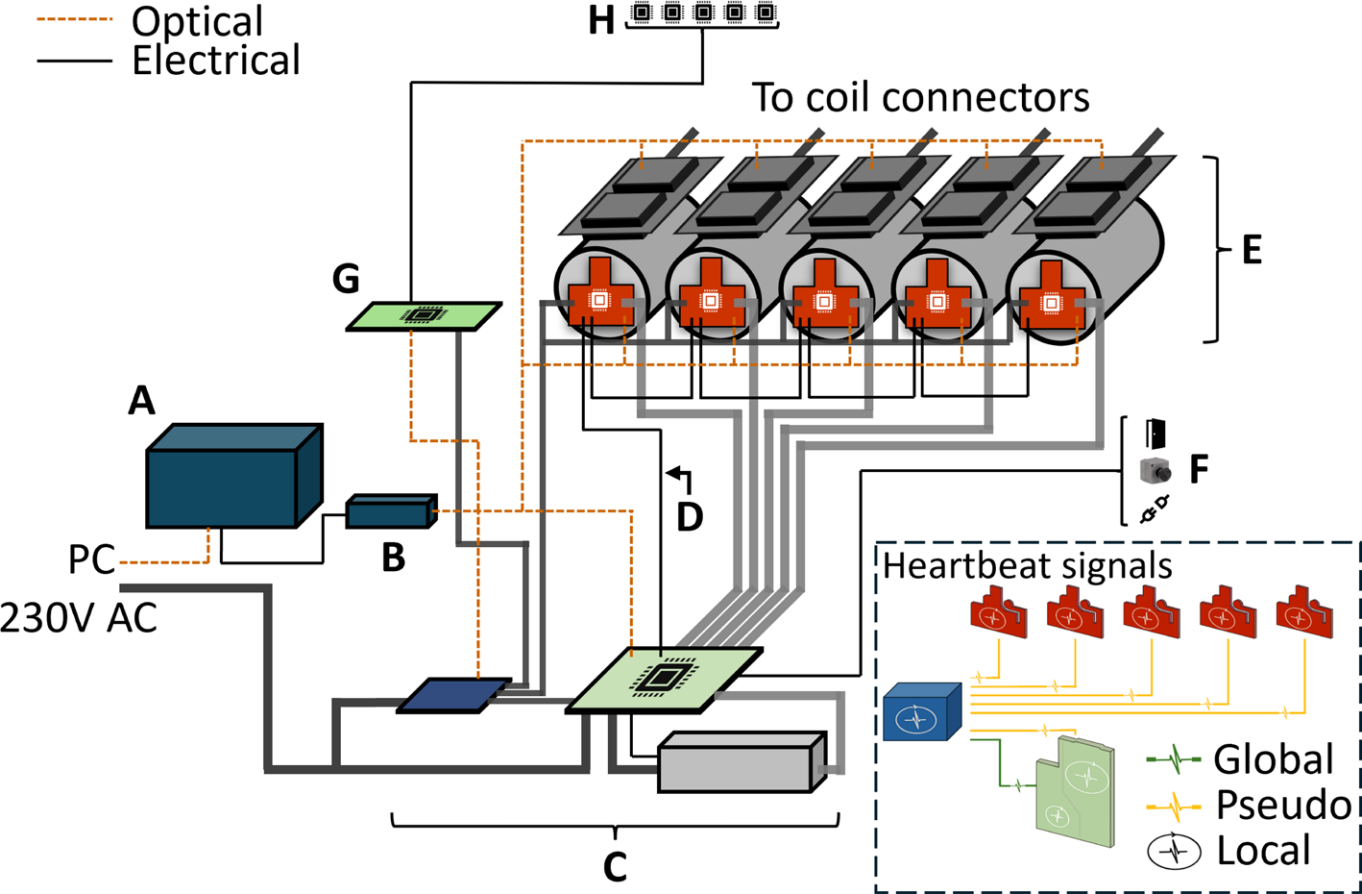
The internal modules of the mTMS device and their connectivity. The modules that contain a microcontroller are marked with a microcontroller symbol. **A** control unit, **B** optical conversion, **C** DC power distribution (blue board), charger interface and safety monitor (green board), high-voltage charger (gray box), **D** safety bus, **E** channels, consisting of the pulse capacitors (gray cylinders), discharge controllers (red boards), and H-bridges (dark gray), **F** door switches, emergency button, and coil connectivity indicator, **G** sensor interface, **H** coil-specific memories and sensors. The inset shows local and global heartbeat signals

The mTMS device is internally controlled with a field-programmable gate array (FPGA; Kintex-7 160 T; Xilinx Inc, USA) housed in a dedicated chassis (PXIe-7820R; National Instruments Corp, USA). The digital input and output signals of the FPGA pass through an electrical-to-optical converter to isolate the FPGA from the high-voltage circuitry. The FPGA and the chassis form the control unit, which is connected to every circuit board in the system. It directly commands the operation of the mTMS device, based on requests from the PC. The connectivity between the modules is presented in Fig. 4.

The stimulation pulses are generated in separate power circuits, each consisting of an H-bridge and a pulse capacitor (960 µF, DCHPS06960EW00JS0F; WIMA GmbH & Co KG, Germany). Insulated-gate bipolar transistors (IGBTs; 5SNA 1500E330305; ABB Power Grids Switzerland Ltd, Switzerland) serve as switching elements. Together, the H-bridge and the capacitor form a channel (Fig. 3B), into which a stimulation coil can be connected via a robust TMS connector (Nexstim Plc, Finland). Each channel includes a resistor (1 kΩ, TE1000B1K0J; TE Connectivity Ltd, Switzerland) for discharging the capacitor in a controlled manner. The pulse capacitors are charged to the desired voltages with a high-voltage charger (CCPF-1500; Lumina Power Inc, USA), which is shared between the channels. The maximum voltage of the charger is 1500 V.

The device power is delivered via an isolation transformer (3000 VA, KKSA-3500; Muuntosähkö Oy, Finland), located at the bottom of the cabinet. The power input is fuse-protected and filtered with an off-the-shelf component; the transformer output is directly fed into the control unit, several power distribution modules, and the high-voltage charger.

### Circuit board modules

The device is divided on the level of circuit boards into programmable and nonprogrammable modules (Fig. 4). The programmable modules, except for the control unit, are managed by PIC microcontroller units (Microchip Technology Inc, USA). The nonprogrammable modules have rudimentary logic and safeguards in hardware but contain no microcontrollers.

*The discharge controllers* are circuit boards responsible for monitoring the voltages of the pulse capacitors and discharging them. Each of the five pulse capacitors has a dedicated discharge controller. The circuit board contains impulse resistors (AY470KE; Ohmite Manufacturing Co LLC, USA) to rapidly discharge the pulse capacitor with a time constant of 80 ms in case of a device malfunction. Separate supervision circuitry, implemented on the discharge controller, tracks the proper operation of the discharge controller’s MCU with a board-level heartbeat signal. Each board can trigger an emergency signal, which is broadcast to the other modules. The emergency signal can be triggered by the microcontroller, the supervision circuitry, or a dedicated safety bus, described in Section D.

*The charger interface* monitors and controls the high-voltage charging unit and selects the pulse capacitor to be charged. The hardware physically restricts the charger from connecting simultaneously to multiple capacitors in case of a firmware error. Similarly to the discharge controllers, a separate board-level supervision circuitry monitors that the MCU operates properly.

*The safety monitor* monitors the device state and contains hardware to detect and respond to errors and malfunctions^1^ by initiating emergency procedures. A separate board-level supervision circuitry monitors the status of the on-board MCU. Additionally, a global heartbeat is transmitted between the control unit and the safety monitor. The safety monitor is implemented on the same circuit board as the charger interface.

*The sensor and memory interface* detects and communicates with external sensors and memories. These include temperature sensors (DS18B20; Maxim Integrated Products Inc, USA) inside the coil array and a dedicated memory unit accompanying each coil. The type of temperature sensor was selected due to its ability to operate with a single data line. In addition, the microcontroller on the circuit board of the interface contains memory to store system-wide parameters.

The nonprogrammable modules provide the supporting framework for the device:

*Electrical-to-optical converter* converts electrical signals to optical and vice versa. This conversion provides galvanic isolation and noise immunity, making the connections between the high-voltage components and the control unit safe.

*Power distribution* handles the controlled and timely distribution of DC power within the system. This allows, for instance, starting and stopping the device in a controlled manner.

*Drive segment* handles the turn-on and turn-off procedures of the IGBTs and provides feedback of their states. The drive segment consists of the individual driver boards, one for each IGBT. These boards set the H-bridge states in each channel, thus controlling pulse generation.

*Trigger module* provides galvanically isolated connectivity between the mTMS device and external instruments. This enables sending trigger signals to other devices, such as an EEG device or another TMS device. The trigger module can also relay trigger signals from external sources.

**Internal communication** The microcontrollers communicate directly with the control unit via galvanically isolated pathways, provided by the electrical-to-optical converter. The communication uses universal asynchronous receiver–transmitter (UART) protocol [42], as it is simple to adapt to optical communication.

### Safety

To accomplish a safe mTMS device, we designed the system to predict the outcomes of various actions and prevent those with potentially hazardous outcomes. To further ensure safety, the design was robustified by implementing redundancy in both hardware and software. In addition, the device was designed to continuously monitor its state to ensure that the device operates within the specifications.

An example of predicting an outcome is the charging of a capacitor: the estimated charging time can be compared to the actual charging duration. A large difference between the two indicates a malfunction. However, some events cannot be predicted, such as the cabinet door being opened while the device is powered. This could happen, for instance, during maintenance. Hazards in such cases are prevented with locks and door status switches.

Redundancy was implemented in the circuit board design and firmware. This was done in particular to counteract hardware and software failures, such as programming errors and circuit-level failures or defects. Redundancy of the design extends beyond the software and the physical implementation, also covering the flow and processing of information within the system.

We designed the hardware to be inherently at least single-fault safe, as stipulated by the standard for electrical safety of medical devices IEC 60601-1 [43]. Consequently, two or more independent failures must occur simultaneously for a malfunction to pose a safety risk.

In addition to technical means of protection, the human element was taken into account. This includes increasing general awareness of how to use the system, the risks involved, and how the device changes the existing workflow in the clinical environment. Due to the experimental nature of the present device, the researchers and medical professionals undergo training before being allowed to operate the system.

**State tracking** The core principle in making the device safe is state tracking, meaning that the control unit evaluates the overall system state based on the information supplied by the individual modules. If the state is unknown, proper operation cannot be ensured. In such situations, the device is immediately disarmed. Several design decisions support the principle of state tracking: redundant design, distributed safety checks, and carefully designed connectivity between modules. In addition, various mechanisms on the hardware level support state tracking.

**Safety bus** The connectivity between the modules is centralized: all circuit boards communicate directly with the control unit (Fig. 4). Additionally, a pathway for safety-related information, safety bus, connects the discharge controllers and the safety monitor. The bus allows direct exchange of information between the boards, enabling each circuit board to read the system state from the bus and to report an emergency. As the information flows directly between the boards, they can respond rapidly to malfunctions and safety–critical events. Any error reported on the safety bus results in an emergency shutdown and prevents the device from restarting. In addition, the safety bus increases redundancy in the system.

**Heartbeat signals** The still-alive status of the device is tracked with periodic heartbeat messages transmitted between and within modules. The response to the heartbeat is monitored to ensure that all modules are still operational. In addition, the delay of the response must lie within a module-specific tolerance.

The heartbeats are divided into three categories: (1) a global heartbeat is transmitted between the control unit and the safety monitor; (2) on the level of circuit boards, the microcontrollers transmit local heartbeat signals to their respective supervisor circuits; (3) periodic status requests and updates are transmitted to and from the circuit boards. A failure to respond in time to any of these signals results in a system-wide emergency shutdown, disarming the device. The different heartbeat signals are presented in Fig. 4.

**Device maintenance** As the mTMS device operates with capacitor voltages of up to 1500 V, it is imperative to restrict access to the cabinet interior. To this end, several safeguards were implemented.

As mandated by IEC 60601-1 [43], a tool—in our case, a key to unlock the cabinet doors or a screwdriver to unmount the side panels—is needed to access the cabinet’s internal electronics (Fig. 3A), including the control unit. For added safety, the state of the door switches is tracked by the safety monitor, initiating an emergency shutdown if either of the two doors is opened.

**Emergency button** An emergency button is mounted on the cabinet wall to enable a manual emergency shutdown.

**Coil connectivity** The coils are connected to robust connectors, which are mounted on the cabinet wall. Each coil connector has an adjacent slot for a connector cover, which is a plastic structure covering the connector (Fig. 3A). The cover physically prevents the respective coil from being connected or disconnected while the device is operational. The mTMS device has higher-capacity pulse capacitors (960 µF) than typical commercial TMS devices, resulting in longer discharge times. Each cover is electrically connected to the safety monitor, triggering an emergency shutdown if a cover is missing. The shutdown rapidly discharges the pulse capacitors, preventing a risk of exposure to high voltages.

If any coil connector does not have a coil attached, the device cannot be started. Coil arrays with fewer than five coils can be used by connecting custom blockers to the remaining coil connectors. Each coil contains a small memory unit, which stores coil-specific parameters and a unique coil identifier. The identifier is read from each channel during start-up. If a coil identifier is incorrect or missing, the start-up is prevented.

The coil array contains integrated temperature sensors for monitoring and estimating the surface temperature at multiple locations. The mTMS device supports at most one sensor for each coil connector. The temperature sensors can be used to model the temperature behavior of the coil array's parts in contact with the patient. A reliable model would allow implementing various temperature limits, such as the hard limit of 41 °C, as specified in IEC 60601-1.

**Device start-up** The start-up is designed to avoid the device being powered and operational while a malfunction is present. When powered, the device runs a start-up sequence,^2^ which starts all the modules in a specific order. After a module reports a successful start-up, the control unit proceeds to start the next module in the sequence. In addition to increasing reliability, the well-defined start-up sequence helps troubleshoot errors in the device.

If any step in the start-up sequence fails, the device is shut down and disarmed. After start-up, the device keeps monitoring the heartbeat signals and status updates from the modules. Additionally, the feedback from the IGBTs is continuously monitored while the device is operational.

Similarly to the start-up sequence, the device performs a well-defined shutdown sequence. In it, the high-voltage charger and the H-bridges are first disabled. Next, the pulse capacitors are discharged, the charger is powered off, and the remaining DC power is disabled. Finally, the control unit resets its state.

### System software

The functionality of the device is distributed among the circuit boards. For instance, several modules contain their own microcontrollers, which govern the functions of the module. This reduces the complexity of tasks required from the control unit.

In the design, nonprogrammable hardware is ultimately responsible for safety: the microcontrollers are used in parallel with dedicated emergency circuits that cannot be overridden. Therefore, the microcontrollers can never perform dangerous actions by themselves. Consequently, programming errors are less likely to have hazardous outcomes.

We followed good programming practices [44], including pair programming, regular code reviews, version control, and only releasing tested and known good versions of the software. In addition, we followed defensive programming practices [45], such as safety–critical functions not expecting other parts of the firmware to work correctly. Moreover, safety-related parts of the code were identified and marked.

**Firmware design** The microcontrollers run module-specific firmware, which implements a simple state machine with two states. In the normal state, the microcontroller responds normally to the commands received via UART. In the emergency state, it only responds to queries by reporting its state without performing actions. Once entered, the microcontroller remains in the emergency state indefinitely.

Special attention was paid to correctly handling interrupts. For instance, the functions called by interrupt handlers were designed to be re-entrant, i.e., the function can be re-entered by another interrupt handler while already being executed, and the function will still work correctly.

We wrote a custom UART library in embedded C for communication between the control unit and the microcontrollers. The library provides a messaging protocol, which contains marker bytes for the beginning and the end of a message, and simple error detection using a parity byte. For instance, a corrupted message failing the parity check causes the microcontroller to enter the emergency state.

**Information flow** The device’s logical core is an FPGA, located in the control unit. As an FPGA operates inherently in parallel, it integrates well with the design of a multichannel TMS device, in which the channels must function independently. In our design, the FPGA has two main responsibilities: (1) provide an interface for using the system and (2) ensure that the device works correctly.

The device’s modules transmit information about their states to the FPGA. The FPGA evaluates the overall system state by combining this information. A discrepancy results in an emergency shutdown and system disarm. As the safety–critical modules are interconnected via the safety bus, these modules detect a device malfunction even if the FPGA fails.

The FPGA sends periodic status messages to the control PC via Thunderbolt connection. These messages include each channel’s state and any errors present in the device. The channel state contains the temperature reading, capacitor voltage, and the total pulse count in the respective coil. A two-way heartbeat between the PC and the FPGA ensures that if the PC encounters a critical problem or fault, the mTMS device is disarmed.

**Fig. 5:**
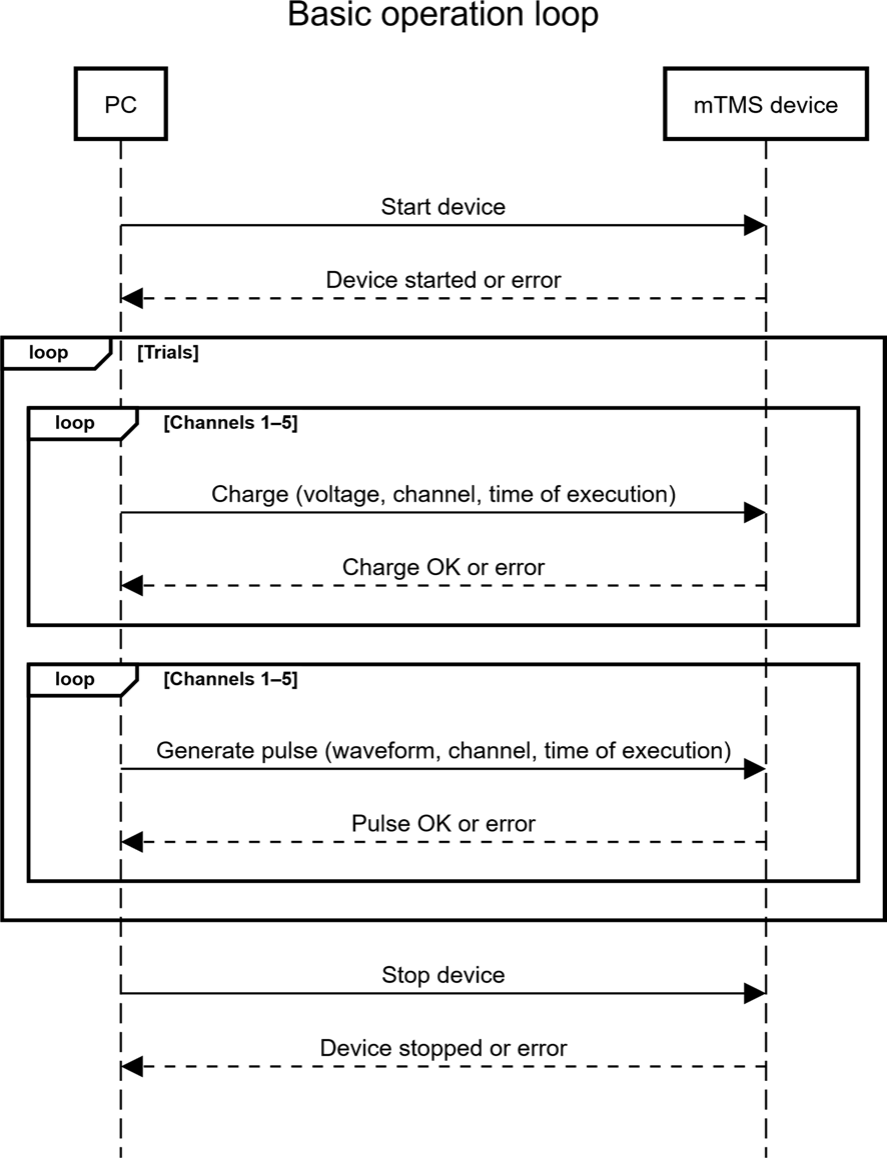
The basic operation loop for controlling the mTMS device. A simple experiment consists of charging each channel to the desired voltage and scheduling a simultaneous pulse at a specific time. This sequence is repeated for the duration of the experiment. Control of triggers is omitted for simplicity

**Action-based control** The FPGA listens to requests from the control PC and executes the corresponding actions. These actions are: (1) driving a TMS pulse with specified waveforms, (2) charging a channel to a given voltage, (3) discharging a channel, and (4) sending a trigger signal. An invalid or infeasible request is disregarded, and an error is returned instead. After the action is executed, a feedback message is sent back to the control PC. The feedback includes the time of execution and whether the action was carried out successfully. Figure 5 illustrates the basic operation loop for controlling the mTMS device.

The control PC runs a program designed specifically for controlling the mTMS device, written in Python and C++. A web user interface was developed in JavaScript with the React library to enable simple stimulation protocols, such as paired-pulse or repetitive TMS (rTMS) to distinct locations on the cortex. For more elaborate stimulation protocols, the device can be controlled with an application programming interface (API) using Python or MATLAB (The MathWorks Inc, USA).

The mTMS device maintains an internal clock, allowing precise timing of actions and synchronization of the device with other instruments. The internal clock, its synchronization logic, and the API are described in detail in [46]. That article also describes additional features of the device, such as using incoming trigger signals to set off one or several actions.

As the device is designed to support various stimulation protocols, the actions can be combined flexibly without rigid safeguards. However, to limit the maximum exposure of the patient to TMS-induced electric fields, the device includes a configurable safeguard, allowing the operator to set the maximum number of stimulation pulses inside a sliding time window. For instance, the safeguard can be used to implement the exposure limits proposed by Rossi et al. [47, 48] to ensure safe use in rTMS. In addition, the safeguard is lenient enough to enable paired-pulse stimulation. Alternative safeguards can be implemented to allow, e.g., theta-burst stimulation and other high-frequency protocols.

### Coil array

We designed and built a coil array, consisting of five overlapping coils. This array allows electronic control of the orientation and location of the electric field maximum induced on the cortex within a 3 cm-diameter region. The coils’ winding paths were obtained using the procedure described in [18].

A mathematical model of a commercial figure-of-eight coil [49] (17 cm × 10 cm; Nexstim Plc, Finland) was used to simulate field patterns inside a spherically symmetric volume conductor. The coil bottom was set outside the sphere, at 85 mm distance from the sphere origin, and the field was computed on a 70 mm radius spherical surface. Different combinations of coil positions and orientations were generated to evenly distribute the locations of the field maximum on a 30-mm-diameter spherical cap. The peak electric field was rotated in 30° steps from 0° to 360° in each location, resulting in 8964 unique combinations of locations and orientations.

**Fig. 6:**
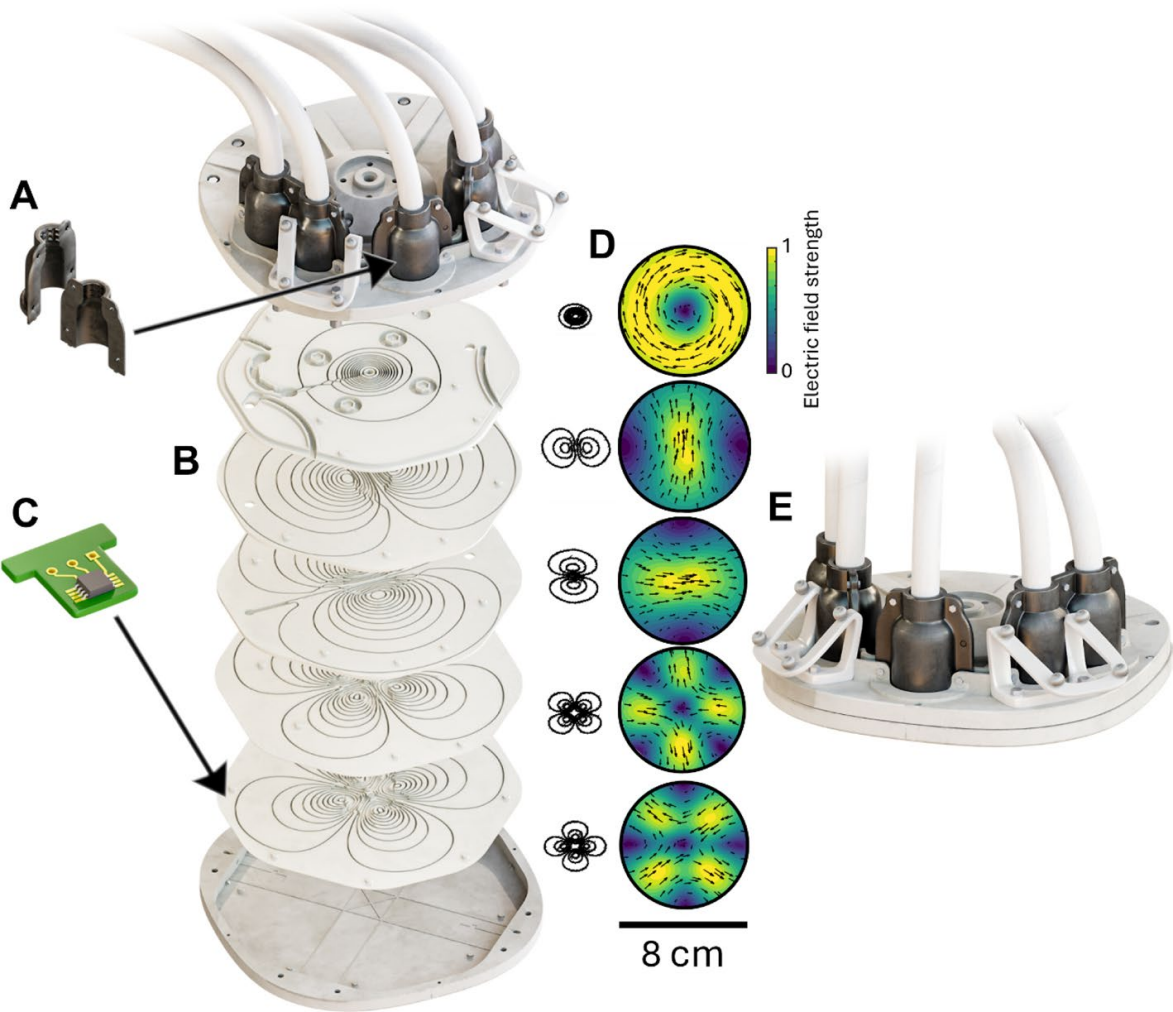
The five-coil array, its parts, winding patterns, and the measured electric field distributions: **A** coil cable holder, **B** coil winding plates, **C** a temperature sensor, **D** coil winding patterns and their normalized electric field patterns on a spherical surface under the array’s center, **E** the complete coil array

Surface current distributions that effectively reproduce the focality and intensity of these electric field patterns were estimated using a custom minimum-energy optimization algorithm on a set of flat planes (distance 85–97 mm from origin). Five basis patterns were obtained from the surface current distributions using singular value decomposition and discretized into coil windings, illustrated in Fig. 6D. Despite the spherically symmetric model, the generated set of coils is suitable for targeting pulses using realistic head models [18].

The coil array consists of an enclosure, five coil-winding plates, navigation trackers, five coil cables, and cable holders. The navigation trackers were designed using an open-source library [50] and manufactured with stereolithographic 3D printing. The coil winding plates, cable holders, and enclosure were designed in Fusion 360 (Autodesk Inc, USA). The winding plates were stacked on top of each other and housed inside the enclosure. The full assembly is illustrated in Fig. 6.

Each plate provides a winding pattern for a TMS coil. The plates were milled from acrylonitrile butadiene styrene (ABS; cwmk GmbH, Germany) due to its good mechanical and thermal properties. The coils were manually wound into the grooves of the winding plates using copper litz wire (1.7 mm diameter, 3-layer Mylar coating, 40 individual strands; Rudolf Pack GmbH & Co KG, Germany). Finally, the plates were press-laminated with a two-component epoxy into a solid, monolithic structure. The wire ends were crimped to a low-inductance, medical-grade cable (Nexstim Plc, Finland). The bottom plate contains three slots for the temperature sensors. Due to dense coil windings at the center, the sensors were placed near the edges of the plate. The coil cables contain separate data lines for sensor data.

The coil cable holders were 3D-printed with Dura resin using stereolithography (Form 3; Formlabs Inc, USA), as it has suitable mechanical durability and flexibility. The cable holders were secured to the enclosure, keeping the coil cables firmly in place and providing strain relief for the cable–litz wire interface.

The enclosure, which is in direct contact with the scalp, was milled from polyoxymethylene (POM) due to its biocompatibility and mechanical and thermal properties. The bottom of the enclosure is 1 mm thick to provide additional mechanical strength and electrical insulation. In addition, there is a 0.5 mm air gap between the bottom of the enclosure and the bottom coil plate, improving the airflow inside the enclosure.

The enclosure, coil winding plates, and coil cable holders were designed to follow the IEC 60601-1 standard for the safety of medical electrical equipment [43]. To provide two means of patient protection with an operating voltage of 1500 V, the creepage and clearance distances were at least 34.0 and 19.2 mm, respectively, between the wires of different coils, the wires and any metallic screws, and the wires and the assembly’s outer surface.

The coil array was characterized by measuring the resistance, self-inductance, and the spatial distribution of the induced electric field of each coil, following [18].

To verify the design and implementation of the coil array, the coil-specific electric fields were measured at 1000 points, uniformly distributed on a 70 mm radius spherical surface, with our custom-built TMS characterizer mimicking a spherically symmetric volume conductor [51]. The distance between the coil array’s bottom center and the sphere origin was 85 mm, matching the model used to design the coils. The measured electric field distributions are visualized in Fig. 6D.

The coil resistances were measured with a 4-wire measurement set-up using a bench multimeter (HP 34401A; Hewlett-Packard Company, USA) and self-inductances with an LCR meter (ELC-130; Escort Instruments Corp, Taiwan).

For each coil, a reference pulse waveform was defined to ensure that no residual currents are left circulating in the power circuit after a pulse. The current was measured with a Rogowski probe (CWT 60B; Power Electronic Measurements Ltd, UK), connected to an oscilloscope (InfiniiVision MSOX3034T; Keysight Technologies Inc, USA).

*.*

### Device testing

The device was tested to ensure intended functionality. First, the individual circuit boards were tested with dedicated test jigs to detect board defects and programming errors. Then, the safety–critical circuit boards were tested on a test bench to verify that the boards operate correctly together. Finally, system-level tests were performed to assess the full functionality of the device.

The jigs automatically tested key features of the circuit boards, minimizing human error. For instance, the jig for the safety monitor and charger interface emulated the start-up process of the high-voltage charger under different conditions. Any deviation from the expected behavior indicated an error.

The system-level tests were performed by verifying that the device operates correctly while gradually increasing connectivity between modules. The components controlling the pulse generation and charging the capacitors were connected and powered last. Connecting modules incrementally allowed safe testing of the device, as high-voltage parts were involved.

After installing the mTMS device, the directions of pulse currents in the coils were verified with a round search coil (30 mm radius) and an oscilloscope (InfiniiVision MSOX3034T).

### Experimental workflow

**Fig. 7:**
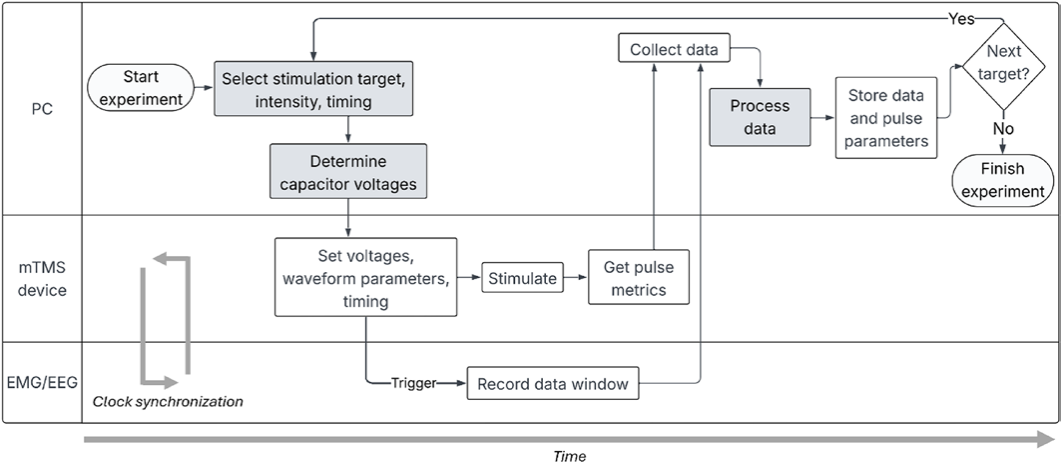
Experimental workflow. An experiment is performed by a script executing a loop on the control PC. The dark gray boxes represent flexible code blocks, allowing the user to implement their own algorithms and methods. During the experiment, the mTMS device waits for instructions from the PC while maintaining clock synchronization with the EMG/EEG device

In contrast to conventional TMS devices, the workflow for performing experiments is different with the mTMS system. An experiment with the device consists of a script running on the mTMS control PC, executing a simple loop (Fig. 7) with three main tasks: (1) selecting a stimulation target, (2) determining the capacitor voltages, and (3) processing the acquired data.

In a simple experiment, the capacitor voltages corresponding to the desired stimulation target can be fetched from a look-up table when using a spherical cortex model for targeting [26]. At its simplest, the data processing step can consist of checking the validity of the measured data and that pulses have been delivered with the intended parameters.

In a more complex experiment, the stimulation target can be calculated based on the acquired response data from previous stimuli [29, 30, 52], and the capacitor voltages determined by modeling the E-field on a realistic cortex and head model [53]. In addition, an algorithm calculating pulse-width modulated waveforms can be applied in the voltage determination step to achieve rapid multi-pulse delivery [22]. Furthermore, the timing of the pulse delivery can also be determined based on predicted EEG behavior [54]. Processing the data of such a complex experiment would include applying one or more algorithms that analyze the evoked responses and make decisions based on them.

The experimental block finishes when there are no more stimulation targets to iterate over. During the experiment, the mTMS device waits for instructions from the PC while maintaining clock synchronization with the EMG/EEG device to guarantee timely pulse delivery. The neuronavigation and robot control work independently in parallel with the experimental script, confirming the correct positioning of the coil array before pulse delivery.

### Verification of multicoil targeting

The technical functionality of the device was tested in two of the authors who were healthy, right-handed males (25–35 years), with the primary purpose of demonstrating the electronic targeting of stimulation. We moved and rotated the peak electric field electronically on a grid, located over the left primary motor cortex. The TMS pulses were trapezoidal with phase durations of 60 µs (rise), 30 µs (hold) and 37–44 µs (fall), with the fall-phase duration depending on the coil inductance [18].

This preliminary testing in two subjects (or even up to five subjects) is not considered a study at the Medical Faculty of Tübingen University and is thus exempted from the requirements of ethics committee approval and provision of informed consent, provided that the technical safety of the experimental device had been approved prior to experimentation in humans. The safety of the electronics was approved by an external company (SENECA Medizintechnische Beratungsgesellschaft mbH, Germany; IEC 62353: protective conductor resistance, device leakage current, line voltage) after the deployment of the device. The two included participants were researchers of the “*Brain Networks & Plasticity*” laboratory in Tübingen, and they were fully informed about the mTMS device and the purpose of this initial testing. The testing was carried out in accordance with the declaration of Helsinki. For testing more participants, we have in the meantime obtained approval by the ethics committee of the Medical Faculty of Tübingen (project number 724/2024MP1).

#### Preparation

T1-weighted magnetic resonance images (MRI) were obtained from both subjects for neuronavigation and to facilitate finding the primary motor cortex. To record muscle responses, surface electrodes were attached to the first dorsal interosseous (FDI) muscle in a belly–tendon montage. The skin was prepared by scraping it with abrasive tape to remove nonconducting skin layers, after which it was cleaned with rubbing alcohol. We ensured that the baseline noise level was below 10 µV peak-to-peak (pp) by adjusting the electrodes, their wiring, and external electrical connections.

#### Procedure

By delivering single pulses with a figure-of-eight coil, we determined the coil array’s location and orientation that elicited the largest motor-evoked potential (MEP) amplitudes in the FDI muscle, corresponding to the motor hotspot in the cortex. The pulses were delivered to the contralateral hemisphere. We defined the resting motor threshold (RMT) as the minimum stimulation intensity that elicits MEPs with amplitude over 50 µVpp in at least 50% of the trials [55] and measured it at the hotspot. The robot was used to position and hold the coil array over the hotspot. In this location, we defined a 28 mm × 28 mm rectangular grid of cortical stimulation targets with a spacing of two millimeters (225 points total). For each target, the orientation of the peak electric field was set to match the optimal orientation found for the hotspot. The coil currents required to electronically shift and rotate the peak electric field were computed with a least-squares solver. The grid was placed 15 mm below the coil bottom, on a spherical surface of 70-mm radius, matching the model used to generate the coil windings.

We stimulated each grid location in a pseudo-random order three times (675 pulses total) with an intensity of 110% RMT. The interstimulus interval (ISI) was sampled from a uniform distribution between 3.5–4.5 s. After each 8 min of stimulation, the subject had 5 min of rest.

We generated a heatmap of the MEP amplitudes recorded on the grid and calculated their weighted centroid [55]. Then, we physically moved the coil array on the scalp past the centroid (by 9 and 8 mm for the two subjects, respectively) and locked the coil array in place using the robotic arm. We repeated the grid stimulation at this new location, while maintaining the previously obtained optimal electric field orientation. We generated a second heatmap of the MEP amplitudes obtained in the new location and calculated their weighted centroid. Finally, while maintaining the physical location of the coil array, we electronically targeted the new centroid with pulses (110% RMT) that were rotated in 30° steps to cover a full circle, with 10 pulses per orientation. We generated an orientation-response graph from these MEP responses.

## Supplementary Information

Additional file 1.

Additional file 2.

## Data Availability

The mapping data presented in this study is available on a reasonable request from the corresponding author.

## Footnotes

1 See supplementary material for a list of error types.

2 The start-up sequence is described in detail in the supplementary material.

## Code availability

The mapping data related code is available on a reasonable request from the corresponding author.

## References

[1] Barker AT, Jalinous R, Freeston IL. Non-invasive magnetic stimulation of human motor cortex. The Lancet. 1985. 10.1016/S0140-6736(85)92413-4. 10.1016/s0140-6736(85)92413-4 2860322

[2] Weise K, Numssen O, Kalloch B, Zier AL, Thielscher A, Haueisen J, et al. Precise motor mapping with transcranial magnetic stimulation. Nat Protoc. 2023. 10.1038/s41596-022-00776-6. 10.1038/s41596-022-00776-6 36460808

[3] Groppa S, Oliviero A, Eisen A, Quartarone A, Cohen LG, Mall V, et al. A practical guide to diagnostic transcranial magnetic stimulation: report of an IFCN committee. Clin Neurophysiol. 2012. 10.1016/j.clinph.2012.01.010. 10.1016/j.clinph.2012.01.010 PMC4890546 22349304

[4] Picht T, Mularski S, Kuehn B, Vajkoczy P, Kombos T, Suess O. Navigated transcranial magnetic stimulation for preoperative functional diagnostics in brain tumor surgery. Neurosurgery. 2009. 10.1227/01.NEU.0000348009.22750.59. 10.1227/01.NEU.0000348009.22750.59 19935007

[5] Lefaucheur JP, Picht T. The value of preoperative functional cortical mapping using navigated TMS. Neurophysiol Clin. 2016. 10.1016/j.neucli.2016.05.001. 10.1016/j.neucli.2016.05.001 27229765

[6] Krieg SM. Navigated transcranial magnetic stimulation in neurosurgery. Cham: Springer International Publishing; 2017.

[7] O’Reardon JP, Solvason HB, Janicak PG, Sampson S, Isenberg KE, Nahas Z, et al. Efficacy and safety of transcranial magnetic stimulation in the acute treatment of major depression: a multisite randomized controlled trial. Biol Psychiatry. 2007. 10.1016/j.biopsych.2007.01.018. 10.1016/j.biopsych.2007.01.018 17573044

[8] Galhardoni R, Correia GS, Araujo H, Yeng LT, Fernandes DT, Kaziyama HH, et al. Repetitive transcranial magnetic stimulation in chronic pain: a review of the literature. Arch Phys Med Rehabil. 2015. 10.1016/j.apmr.2014.11.010. 10.1016/j.apmr.2014.11.010 25437106

[9] Ferreri F, Pasqualetti P, Määttä S, Ponzo D, Ferrarelli F, Tononi G, et al. Human brain connectivity during single and paired pulse transcranial magnetic stimulation. Neuroimage. 2011. 10.1016/j.neuroimage.2010.07.056. 10.1016/j.neuroimage.2010.07.056 20682352

[10] Breveglieri R, Borgomaneri S, Filippini M, De Vitis M, Tessari A, Fattori P. Functional connectivity at rest between the human medial posterior parietal cortex and the primary motor cortex detected by paired-pulse transcranial magnetic stimulation. Brain Sci. 2021. 10.3390/brainsci11101357. 10.3390/brainsci11101357 PMC8534070 34679421

[11] Oathes DJ, Duprat RJP, Reber J, Liang X, Scully M, Long H, et al. Non-invasively targeting, probing and modulating a deep brain circuit for depression alleviation. Nat Ment Health. 2023. 10.1038/s44220-023-00165-2. 10.1038/s44220-023-00165-2 PMC12919846 41726164

[12] Cash RFH, Weigand A, Zalesky A, Siddiqi SH, Downar J, Fitzgerald PB, et al. Using brain imaging to improve spatial targeting of transcranial magnetic stimulation for depression. Biol Psychiatry. 2021. 10.1016/j.biopsych.2020.05.033. 10.1016/j.biopsych.2020.05.033 32800379

[13] Menon V. Large-scale brain networks and psychopathology: a unifying triple network model. Trends Cogn Sci. 2011. 10.1016/j.tics.2011.08.003. 10.1016/j.tics.2011.08.003 21908230

[14] Lancaster JL, Narayana S, Wenzel D, Luckemeyer J, Roby J, Fox P. Evaluation of an image-guided, robotically positioned transcranial magnetic stimulation system. Hum Brain Mapp. 2004. 10.1002/hbm.20041. 10.1002/hbm.20041 PMC6872115 15202111

[15] Richter L, Matthäus L, Schlaefer A, Schweikard A. Fast robotic compensation of spontaneous head motion during Transcranial Magnetic Stimulation (TMS). IET Seminar Digest. 2010. 10.1049/ic.2010.0396.

[16] Goetz SM, Kozyrkov IC, Luber B, Lisanby SH, Murphy DLK, Grill WM, et al. Accuracy of robotic coil positioning during transcranial magnetic stimulation. J Neural Eng. 2019. 10.1088/1741-2552/ab2953. 10.1088/1741-2552/ab2953 PMC7297297 31189147

[17] Koponen LM, Nieminen JO, Ilmoniemi RJ. Multi-locus transcranial magnetic stimulation—theory and implementation. Brain Stimul. 2018. 10.1016/j.brs.2018.03.014. 10.1016/j.brs.2018.03.014 29627272

[18] Nieminen JO, Sinisalo H, Souza VH, Malmi M, Yuryev M, Tervo AE, et al. Multi-locus transcranial magnetic stimulation system for electronically targeted brain stimulation. Brain Stimul. 2022. 10.1016/j.brs.2021.11.014. 10.1016/j.brs.2021.11.014 PMC8807400 34818580

[19] Nieminen JO, Koponen LM, Mäkelä N, Souza VH, Stenroos M, Ilmoniemi RJ. Short-interval intracortical inhibition in human primary motor cortex: a multi-locus transcranial magnetic stimulation study. Neuroimage. 2019. 10.1016/j.neuroimage.2019.116194. 10.1016/j.neuroimage.2019.116194 31525495

[20] Sinisalo H, Nieminen J, Ilmoniemi R. Waveform simulation and pulse-width-modulation approximations with multi-locus TMS. Brain Stimul. 2021. 10.1016/j.brs.2021.10.149.

[21] Ilmoniemi R, Nieminen J, Koponen L. Control of transcranial magnetic stimulation. 11,167,147. San Francisco: Google Patents; 2021.

[22] Sinisalo H, Laine M, Nieminen JO, Souza VH, Matsuda RH, Soto AM, et al. Multi-locus transcranial magnetic stimulation with pulse-width modulation. Brain Stimul. 2025. 10.1016/j.brs.2025.04.014. 10.1016/j.brs.2025.04.014 40239740

[23] Souza VH, Nieminen JO, Tugin S, Koponen LM, Baffa O, Ilmoniemi RJ. TMS with fast and accurate electronic control: measuring the orientation sensitivity of corticomotor pathways. Brain Stimul. 2022. 10.1016/j.brs.2022.01.009. 10.1016/j.brs.2022.01.009 35038592

[24] Tugin S, Souza VH, Nazarova MA, Novikov PA, Tervo AE, Nieminen JO, et al. Effect of stimulus orientation and intensity on short-interval intracortical inhibition (SICI) and facilitation (SICF): a multi-channel transcranial magnetic stimulation study. PLoS ONE. 2021. 10.1371/journal.pone.0257554. 10.1371/journal.pone.0257554 PMC8457500 34550997

[25] Matsuda RH, Souza VH, Marchetti TC, Soto AM, Kahilakoski OP, Laine M, et al. Characterizing an electronic–robotic targeting platform for precise and fast brain stimulation with multi-locus transcranial magnetic stimulation. BioRxiv. 2024. 10.1101/2024.03.12.584601. 10.1088/1361-6560/adf36e 40701171

[26] Matsuda RH, Souza VH, Marchetti TC, Soto AM, Kahilakoski O-P, Zhdanov A, et al. Robotic–electronic platform for autonomous and accurate transcranial magnetic stimulation targeting. Brain Stimul. 2024;17:469–72. 10.1016/j.brs.2024.03.022. 38582491 10.1016/j.brs.2024.03.022

[27] Souza VH, Nieminen JO, Tugin S, Koponen LM, Ziemann U, Baffa O, et al. Probing the orientation specificity of excitatory and inhibitory circuitries in the primary motor cortex with multi-channel TMS. Clin Neurophysiol. 2025;169:23–32. 10.1016/j.clinph.2024.11.004. 39603156 10.1016/j.clinph.2024.11.004

[28] Souza VH, Sinisalo H, Korhonen JT, Paasonen J, Nyrhinen M, Nieminen JO, et al. Multi-coil TMS for preclinical applications in ultra-high-field MRI. Imaging Neuroscience. 2025. 10.1162/imag_a_00558. 10.1162/imag_a_00558 PMC12319963 40800998

[29] Tervo AE, Metsomaa J, Nieminen JO, Sarvas J, Ilmoniemi RJ. Automated search of stimulation targets with closed-loop transcranial magnetic stimulation. Neuroimage. 2020. 10.1016/j.neuroimage.2020.117082. 10.1016/j.neuroimage.2020.117082 32593801

[30] Tervo AE, Nieminen JO, Lioumis P, Metsomaa J, Souza VH, Sinisalo H, et al. Closed-loop optimization of transcranial magnetic stimulation with electroencephalography feedback. Brain Stimul. 2022. 10.1016/j.brs.2022.01.016. 10.1016/j.brs.2022.01.016 PMC8940636 35337598

[31] Roth Y, Levkovitz Y, Pell GS, Ankry M, Zangen A. Safety and characterization of a novel multi-channel TMS stimulator. Brain Stimul. 2014. 10.1016/j.brs.2013.09.004. 10.1016/j.brs.2013.09.004 24529836

[32] Navarro de Lara LI, Daneshzand M, Mascarenas A, Paulson D, Pratt K, Okada Y, et al. A 3-axis coil design for multichannel TMS arrays. Neuroimage. 2021. 10.1016/j.neuroimage.2020.117355. 10.1016/j.neuroimage.2020.117355 PMC7837414 32916290

[33] Matsubara T, Daneshzand M, Navarro de Lara L, Sundaram P, Hämäläinen M, Stufflebeam S, et al. A 16-channel transcranial magnetic stimulation (TMS) system: its magnetic field and induced electric field patterns. Brain Stimul. 2023. 10.1016/j.brs.2023.01.402.

[34] Luber B, Davis SW, De DZ, Murphy D, Martella A, Peterchev AV, et al. Using diffusion tensor imaging to effectively target TMS to deep brain structures. Neuroimage. 2022. 10.1016/j.neuroimage.2021.118863. 10.1016/j.neuroimage.2021.118863 PMC8851689 34974116

[35] Aydogan DB, Souza VH, Matsuda RH, Lioumis P, Ilmoniemi RJ. Real-time tractography-assisted neuronavigation for transcranial magnetic stimulation. Hum Brain Mapp. 2025. 10.1002/hbm.70122. 10.1002/hbm.70122 PMC11685379 39737576

[36] Souza VH, Matsuda RH, Peres ASC, Amorim PHJ, Moraes TF, Silva JVL, et al. Development and characterization of the InVesalius navigator software for navigated transcranial magnetic stimulation. J Neurosci Method. 2018. 10.1016/j.jneumeth.2018.08.023. 10.1016/j.jneumeth.2018.08.023 30149047

[37] Marinetto E, Garcia-Mato D, Garcia A, Martinez S, Desco M, Pascau J. Multicamera optical tracker assessment for computer aided surgery applications. IEEE Access. 2018. 10.1109/ACCESS.2018.2878323.

[38] Sweeney KT, Ward TE, McLoone SF. Artifact removal in physiological signals-practices and possibilities. IEEE Trans Inf Technol Biomed. 2012. 10.1109/TITB.2012.2188536. 10.1109/TITB.2012.2188536 22361665

[39] Ilmoniemi RJ, Hernandez-Pavon JC, Makela NN, Metsomaa J, Mutanen TP, Stenroos M, et al. Dealing with artifacts in TMS-evoked EEG. Proceedings of the annual international conference of the IEEE engineering in medicine and biology society, EMBS, 2015. 10.1109/EMBC.2015.7318342. 10.1109/EMBC.2015.7318342 26736242

[40] Urigüen JA, Garcia-Zapirain B. EEG artifact removal—state-of-the-art and guidelines. J Neural Eng. 2015. 10.1088/1741-2560/12/3/031001. 10.1088/1741-2560/12/3/031001 25834104

[41] Nyrhinen MJ, Souza VH, Ilmoniemi RJ, Lin FH. Acoustic noise generated by TMS in typical environment and inside an MRI scanner. Brain Stimul. 2024. 10.1016/j.brs.2024.02.006. 10.1016/j.brs.2024.02.006 38342363

[42] Axelson J. Serial port complete: COM ports, USB virtual COM ports, and ports for embedded systems. Chicago: Lakeview Research; 2007.

[43] Standard 11266 I. IEC 60601-1. 61010-1 © Iec:2001 2014;2014.

[44] Martin RC. Clean code: a handbook of agile software craftsmanship. Kybernetes. 2009. 10.1108/03684920910973252.

[45] Stueben M. Good habits for great coding: improving programming skills with examples in python. Singapore: Springer; 2018.

[46] Kahilakoski O-P, Sinisalo H, Nieminen JO, Alkio K, Valén K, Kozák G, et al. A high-precision timing method and digital interface for closed-loop TMS. New York: Cold Spring Harbor Laboratory; 2025.

[47] Rossi S, Hallett M, Rossini PM, Pascual-Leone A, Avanzini G, Bestmann S, et al. Safety, ethical considerations, and application guidelines for the use of transcranial magnetic stimulation in clinical practice and research. Clin Neurophysiol. 2009. 10.1016/j.clinph.2009.08.016. 10.1016/j.clinph.2009.08.016 PMC3260536 19833552

[48] Rossi S, Antal A, Bestmann S, Bikson M, Brewer C, Brockmöller J, et al. Safety and recommendations for TMS use in healthy subjects and patient populations, with updates on training, ethical and regulatory issues: expert guidelines. Clin Neurophysiol. 2021. 10.1016/j.clinph.2020.10.003. 10.1016/j.clinph.2020.10.003 PMC9094636 33243615

[49] Ueno S, Tashiro T, Harada K. Localized stimulation of neural tissues in the brain by means of a paired configuration of time-varying magnetic fields. J Appl Phys. 1988. 10.1063/1.342181.

[50] Brown A, Uneri A, De ST. Design and validation of an open-source library of dynamic reference frames for research and education in optical tracking. J Med Imag. 2018. 10.1117/1.jmi.5.2.021215. 10.1117/1.JMI.5.2.021215 PMC5806031 29487887

[51] Nieminen JO, Koponen LM, Ilmoniemi RJ. Experimental characterization of the electric field distribution induced by TMS devices. Brain Stimul. 2015. 10.1016/j.brs.2015.01.004. 10.1016/j.brs.2015.01.004 25680320

[52] Granö I, Kahilakoski O-P, Kirchhoff M, Ahola O, Pieramico G, Laine M, et al. Targeting multi-locus TMS with Bayesian optimization. Brain Stimul. 2025;18:298. 10.1016/j.brs.2024.12.250.39988120

[53] Stenroos M, Koponen LM. Real-time computation of the TMS-induced electric field in a realistic head model. Neuroimage. 2019. 10.1016/j.neuroimage.2019.116159. 10.1016/j.neuroimage.2019.116159 31494248

[54] Pankka H, Lehtinen J, Ilmoniemi RJ, Roine T. Enhanced EEG forecasting: a probabilistic deep learning approach. Neural Comput. 2025;37:793–814. 10.1162/neco_a_01743. 40030141 10.1162/neco_a_01743

[55] Rossini PM, Burke D, Chen R, Cohen LG, Daskalakis Z, Di Iorio R, et al. Non-invasive electrical and magnetic stimulation of the brain, spinal cord, roots and peripheral nerves: basic principles and procedures for routine clinical and research application: an updated report from an IFCN Committee. Clin Neurophysiol. 2015. 10.1016/j.clinph.2015.02.001. 10.1016/j.clinph.2015.02.001 PMC6350257 25797650

[56] Sakai K, Ugawa Y, Terao Y, Hanajima R, Furubayashi T, Kanazawa I. Preferential activation of different I waves by transcranial magnetic stimulation with a figure-of-eight-shaped coil. Exp Brain Res. 1997. 10.1007/BF02454139. 10.1007/BF02454139 9028772

[57] Liu B, Zou D, Feng L, Feng S, Fu P, Li J. An FPGA-based CNN accelerator integrating depthwise separable convolution. Electronics (Switzerland). 2019. 10.3390/electronics8030281.

[58] Lai YH, Ustun E, Xiang S, Fang Z, Rong H, Zhang Z. Programming and synthesis for software-defined FPGA acceleration: status and future prospects. ACM Trans Reconfigurable Technol Syst. 2021. 10.1145/3469660.

[59] Xiao Y, Park D, Butt A, Giesen H, Han Z, Ding R, et al. Reducing FPGA compile time with separate compilation for FPGA building blocks. Proceedings—2019 international conference on field-programmable technology, ICFPT 2019. 2019. 10.1109/ICFPT47387.2019.00026.

[60] Angepat H, Eads G, Craik C, Chiou D. NIFD: Non-intrusive FPGA debugger debugging FPGA “Threads” for Rapid HW/SW systems prototyping. Proceedings—2010 international conference on field programmable logic and applications, FPL 2010. 2010. 10.1109/FPL.2010.77.

[61] Nurmi S, Karttunen J, Souza VH, Ilmoniemi RJ, Nieminen JO. Trade-off between stimulation focality and the number of coils in multi-locus transcranial magnetic stimulation. J Neural Eng. 2021. 10.1088/1741-2552/ac3207. 10.1088/1741-2552/ac3207 34673563

[62] Sinisalo H, Rissanen I, Kahilakoski OP, Souza VH, Tommila T, Laine M, et al. Modulating brain networks in space and time: multi-locus transcranial magnetic stimulation. Clin Neurophysiol. 2024. 10.1016/j.clinph.2023.12.007. 10.1016/j.clinph.2023.12.007 38184469

