# Supplementary material for: Design, construction, and deployment of a multi-locus transcranial magnetic stimulation system for clinical use
Source: Biomed Eng Online. 2025 May 18;24:61. doi: 10.1186/s12938-025-01393-6 (PMC12085834; doi:10.1186/s12938-025-01393-6)
Supplement: Supplementary file 1 — Additional file 1. [file 12938_2025_1393_MOESM1_ESM.pdf]

Supplementary Table 1. Error types, an example source of the error, and a list of potential causes.

Error type	Source	Potential causes
Channel select	Charging channel selection failing	On-board optical short link broken
Charger	Error in high-voltage charger	Charger output over limit
		Charger temperature over limit
Communication	Unsupported or corrupted message	Loose optical fiber
		Failure in circuit board hardware
		Version mismatch between FPGA and MCU
DC power	DC voltages under threshold	Broken component
		Broken power supply
		Broken on-board regulator
Drive segment	Erroneous feedback from IGBT	IGBT failure
		IGBT driver failure
		Power failure
Emergency	An emergency signal in the safety bus	Emergency stop
		Missing connector cover
		Cabinet door opened
Overvoltage	Capacitor voltage over limit	Malfunctioning high-voltage charger
Start-up	Start-up conditions not met	Emergency error present
		Microcontrollers not starting up
		Residual voltages in pulse capacitors
Synchronization	Missing heartbeat or status message	Nonresponsive control unit
		Nonresponsive circuit board
