# Supplementary material for: Design, construction, and deployment of a multi-locus transcranial magnetic stimulation system for clinical use
Source: Biomed Eng Online. 2025 May 18;24:61. doi: 10.1186/s12938-025-01393-6 (PMC12085834; doi:10.1186/s12938-025-01393-6)
Supplement: Supplementary file 2 — Additional file 2. [file 12938_2025_1393_MOESM2_ESM.pdf]

Supplementary Table 2. The start-up sequence

Step #	Description
1	Enable DC power to the safety monitor, discharge controllers, charger interface, and sensor and memory interface
2	Wait for a start-up message from the safety monitor and the discharge controllers
3	Wait until the safety monitor and the discharge controllers start sending periodic status messages. Request the status of the charger interface.
4	Check that the status messages indicate no errors.
5	Check that each coil connector has a valid coil connected to it.
6	Enable DC power to the channel selector segment of the charger interface.
7	Check that the residual capacitor voltages reported by the discharge controllers are zero.
8	Cross-compare the capacitor voltages reported by the discharge controllers with those reported by the charger interface.
9	Enable DC power for the drive segment. Verify the feedback signals from the IGBTs.
10	Set the H-bridges into a safe default state.
